# Characterization of the complete mitochondrial genome of *Takifugu pseudommus* (Actinopterygii, Tetraodontidae)

**DOI:** 10.1080/23802359.2022.2054739

**Published:** 2022-03-28

**Authors:** Tae Sun Kang

**Affiliations:** Department of Food Science and Technology, Seoul Women’s University, Seoul, South Korea

**Keywords:** Complete mitochondria genome, *Takifugu pseudommus*, Tetraodontidae, phylogenetic analysis

## Abstract

The complete mitochondrial genome of *Takifugu pseudommus* was reported in the present study. It was 16,448 bp in length and consisted of 13 protein-coding genes, two ribosomal RNA genes, 22 transfer RNA genes, and a non-coding control region. The nucleotide content of this genome was 29.91% for A, 29.09% for C, 15.20% for G, and 25.80% for T. The phylogenetic tree, constructed with complete mitochondrial genome, suggested *T. pseudommu*s was closely related with *T. chinensis*, *T. flavidus*, and *T. rubripes* among Tetraodontidae species. This study could provide an important dataset for genetic diversity and species identification among the genus *Takifugu*.

*Takifugu pseudommus* (Chu 1935) is distributed in Northwest Pacific including western and southern coast line of Korea and northern East China Sea (Cui et al. 2005). *Takifugu chinensis* and *T. rubripes* have the same habitat and similar morphological character, frequently leading to disputes for the identification and classification of these three species (Park et al. 2020). In this regard, the complete mitochondrial genome of *T. pseudommus* was characterized, and phylogenetic relationship with pufferfish of the genus *Takifugu* was analyzed first.

*Takifugu pseudommus* was collected from Daejin port, Gangwon-do, South Korea (N 38°30'2", E128°25'37") on 13 January 2016. A specimen was deposited at the National Institute of Fisheries Science (NIFS; https://www.nifs.go.kr/page?id=en_index, Hyeong-soo Kim, ecomarine@korea.kr) under the voucher number NFRDI-FI-TS-0049454. It should be noted that this study was conducted using only muscle tissue of *T. pseudommus* that was kindly obtained from NIFS; thus, ethical approval or other permissions were not required. DNA was extracted from 30 mg of muscle tissue using the DNeasy Blood & Tissue kit (Qiagen, Hilden, Germany). Next-generation sequencing was conducted using Illumina MIGseq platform at Gencube Plus (Seoul, South Korea). Sequencing artifacts and low-quality bases were removed using PE mode of Trimmomatic (ver. 0.39), and the mitochondrial genome was assembled de nove using Getorganelle (ver. 1.7.1a) and then annotated using Mitoz (ver. 2.3). Its complete sequence was deposited in the GenBank (MZ603736.1).

The mitochondrial genome of *T. pseudommus* was a circular DNA of 16,448 bp in length, showing a typical mitogenomic organization and gene order as most pufferfish species (Dou et al. 2019; Huang et al. 2020). This genome contained 13 protein coding genes, two ribosomal RNA genes, 22 transfer RNA (tRNA) genes, and an AT-rich control region (821 bp). Most of the genes were encoded on heavy chain, except for 8 tRNA (Gln, Ala, Asn, Cys, Tyr, Ser, Glu, and Pro) genes and one-protein coding gene (NADH dehydrogenases subunit 6; ND6). The nucleotide composition of this genome was 29.91% for A, 29.09% for C, 15.20% for G, and 25.80% for T. Start codon for the protein-coding genes was ATG with the exception of cytochrome C oxidase subunit I (COX1; GTG). Stop codons for the 13 genes were varied; eight genes was terminated with TAA, while TAG and AGG was used in ND3 and ND6 genes, respectively, and an incomplete stop codon (T–) was observed in COX1, COX2, and ND4 genes. The phylogenetic relationship of *T. pseudommus* and other 14 Tetraodontidae species was analyzed using a maximum likelihood (ML) method of MEGA X software. The ML tree was based on the Kimura 2-parameter model of nucleotide substitution. As shown in Figure 1, *T. pseudommu*s was most closely related with *T. chinensis*, *T. flavidus*, and *T. rubripes* species. This is the first report on mitochondrial genome of *T. pseudommu*s, which can provide an important dataset for genetic diversity and species identification among pufferfish of the genus *Takifugu*.

**Figure 1.**
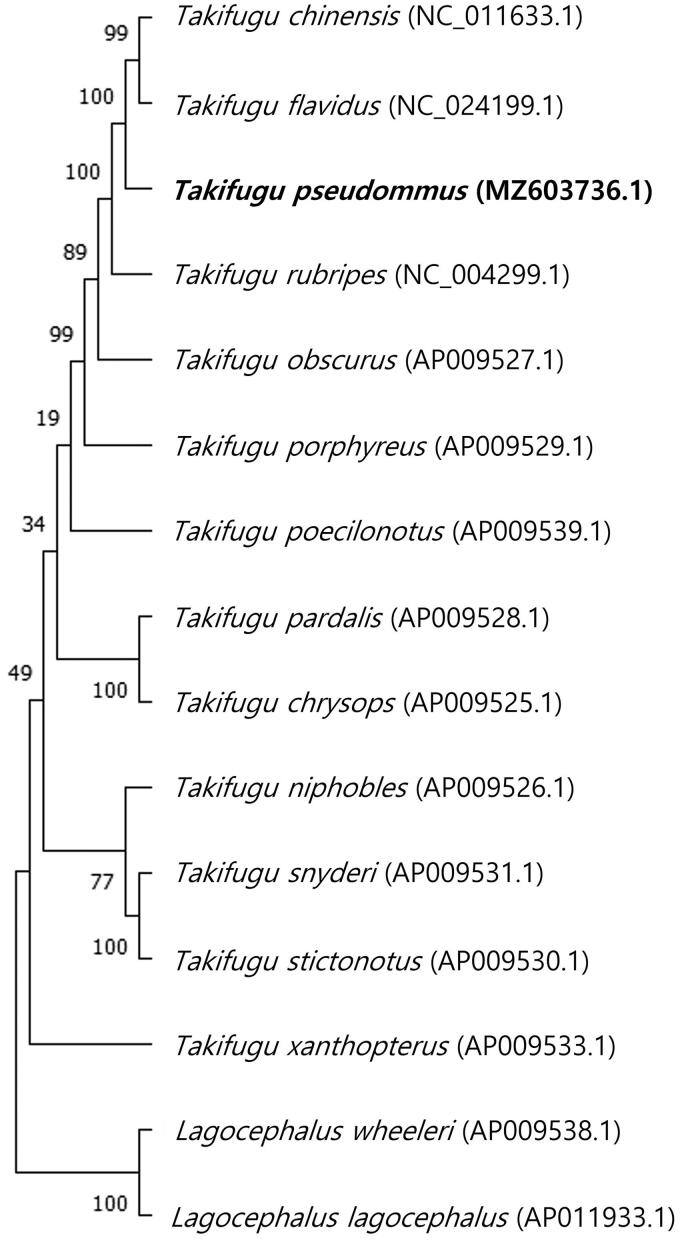
Phylogenetic tree based on complete mitochondrial genome of *Takifugu pseudommus* and other 14 Tetraodontidae species. *Lagocephalus wheeleri* and *L. lagocephalus* were used as outgroup. Numbers at the nodes indicate bootstrap probabilities from 1000 replications.

## Data Availability

The genome sequence data that support the findings of this study are openly available in GenBank of NCBI (https://www.ncbi.nlm.nih.gov/nuccore/mz603736) under the accession no. MZ603736.1. The associated BioProject, SRA, and BioSample numbers are PRJNA792887, SRR17381078, and SAMN24469232 respectively.
